# Buckling analysis of laminated composite elliptical shells using the spline finite strip procedure

**DOI:** 10.1016/j.heliyon.2023.e19328

**Published:** 2023-08-20

**Authors:** Neda Korkeai, As’ad Alizadeh, Davood Poorveis, Shapour Moradi, Pooya Pasha

**Affiliations:** a School of Civil Engineering, Shahid Chamran University of Ahvaz, Ahvaz, 61357, Iran; b Department of Civil Engineering, College of Engineering, Cihan University-Erbil, Erbil, Iraq; c School of Mechanical Engineering, Shahid Chamran University of Ahvaz, Ahvaz, 61357, Iran; d Department of Mechanical Engineering, Mazandaran University of Science and Technology, Babol, Iran

**Keywords:** Composite, Buckling, Elliptical shell, Spline finite strip procedure, First order shear deformation shell theory

## Abstract

According to the first order shear deformation plate assumption, current paper numerically investigates the buckling problem of cylindrical shells with oval cross-section under simply supported boundary condition subjecting to uniform and non-uniform loads via spline finite strip method. Equilibrium governing equations are generated based upon the virtual work's principle. Shell displacement function is assumed to be as dual development of degree 3 spline functions and Lagrange polynomial functions. The analysis method used at any time with any type of loading can calculate deformation and buckling. The step-by-step critical load is calculated using the determinant of the stiffness matrix. In the present paper, it is supposed that the applied linear elastic materials consist of isotropic and non-isotropic materials. Numerical studies are performed to indicate the effects of shell geometry, materials, and layered materials on the buckling load. For the validation purpose, the results of current research are compared with those of previous researches and ABAQUS software.

## 1 Introduction

Elastic instability (buckling) phenomena, caused by applying the axial compressive forces and shearing loads to the structures, is one of the important mechanical characteristics that has always been interested in the design of engineering structures (e.g., beams, plates, tanks, columns, shells) by many researchers [[1], [2], [3], [4], [5], [6], [7]]. Elastic instability (buckling) phenomena could be a shape of flimsiness that happens in a flexible framework, such as buckling of pillars and pieces subjected to compressive loads. Due to the high application demand, shell-type structures, particularly, cylindrical shells having superior mechanical characteristics and excellent high strength performance are applied widely in many engineering problems such as aerospace [8], nuclear reactors [9], the marine industry [10], civil engineering [11], and mechanical engineering [12]. So far, numerous researches investigated the buckling behavior of shell-type structures. For example [13], experimentally and numerically reported the buckling problem of cylindrical shells with longitudinal joint subjected to the lateral external pressure. On the basis of extended Kantorovich method [14], predicated buckling and post-buckling behavior of cylindrical shells under localized external pressure [15]. Numerically studied the effect of periodical imperfections on the changes of the buckling load of closed elastic isotropic shallow conical shells [16]. Used an effective technique for analytically achieving the buckling load of a cylindrical shell under nonuniform external pressure [17]. Carried out the first-order shear deformation in conjunction with Rayleigh–Ritz technique to find the buckling load of the graphene-reinforced porous cylindrical shell.

In recent years, the composite material has been widely used for their superior performance in reinforcing materials and improving specific stiffness, specific strength, resisting and environmental forcing conditions in structural parts. The development of applications using composite materials is because of their desirable features including great specific strength to stiffness ratio, long fatigue life, and magnetic transparency. It should be noted that the realization the relation between fiber orientation, advanced damage, and the corresponding deformation areas is necessary when utilizing such materials in safety-critical applications. The composite cylindrical shells are abundantly employed in the various engineering fields such as aerospace, marine, automobile. So far, many research projects have been carried out focusing on the investigation of the application of composite materials in engineering structures like composite cylindrical and conical shells, [[18], [19], [20], [21], [22], [23], [24], [25]]. Furthermore, recently, buckling problem of composite cylindrical shells are reported by Refs. [[25], [26], [27], [28], [29], [30], [31], [32]]. Fathollahi et al. [33] examined the Investigation of the impact of radiation on 2D MHD Al_2_O_3_ unsteady water stream through parallel compression plate AGM and HPM. The innovation of this study, the dimensionless values of different fluids, such as Prandtel number and coefficient of friction, influence the study of velocity and heat transfer parameters of oxide nanofluids. Shadman et al. [34] studied the combined fins of baffle and chamfer on threatened tension surfaces under the influence of nanofluids and magnetic parameters for rotary joints in computer hardware. The innovation of this paper is to study the parameters of Mwcnt and TiO2 nanofluids transmitted from different fins and blades on the elongated surface and compare their results with each other. Pasha et al. [35] consider the application of numerical techniques to micropolar fluid flow and heat transfer in permeable plates. The novelty of this research is to study heat transfer and fluid flow through two equal sheets with Adomian Decomposition Method (ADM) and Variable Iterative Method (VIM). Low-speed non-linear impact response of a cylindrical porous metal shell reinforced with graphene platelets under axial motion with geometrical imperfections reviewed by Zhang et al. [36,37]. Nonlinear primary resonance analysis of metallic foams reinforced with initial load-bearing graphene platelets, double-curved shells with imperfect geometries examined by Gui-Lin et al. [38]. Katiyar et al. [39] investigated the vibrational response of a functionally classified two-dimensional geometrically discontinuous surface lying on an elastic foundation in a thermal environment with initial imperfections. The preceding review explicitly denoted that there are an increasing number of investigations on the buckling problem of thin-walled composite cylindrical shells with circular cross-section [[40], [41], [42], [43]]. Composite materials are created by combining materials into an overall structure that has distinctive properties than the person components. Composite structures are used in a variety of industries from aerospace, marine, aviation, transportation, sports/recreation to construction [[44], [45], [46], [47]]. However, there seems to be no systematic, analytical or numerical studies on utilizing the FSDT and spline finite strip method (SFSM) to investigate the buckling problem of the laminated composite elliptical cylindrical shell under simply supported boundary condition subjected to the uniform and non-uniform axial loadings. The displacement components are defined as the multiplication expansion of the 3rd degree spline functions in the longitudinal direction and the Lagrange interpolation functions in the crosswise direction. The governing buckling equations are inferred using the vibrational rule and solved with consideration of Sanders nonlinear theory. Analysis was conducted in two steps; in the first step, the shell subjecting to the load and displacements are calculated by static analysis and then the geometric hardness matrix is calculated with respect to Sanders nonlinear theory and FSDT. In the second step, combining the linear and geometric hardness matrices, the critical shell load is calculated by analyzing the special value. At the end, the results inferred from the current analysis are compared with those of previous studies and the results of limited version of the ABAQUS software. The purpose of writing this article and its application in this way is a systematic numerical and analytical study with two new methods, FSDT and SFSM, to investigate the buckling of composite cylindrical shell under a special condition, which is mentioned for the first time in this article. The innovation of this article includes 2 parts related to each other. In the first stage of the new ideation, all displacements and the shell under load were checked with static analysis and the geometric matrix was calculated according to the FSDT theory. In the second stage of innovation in this article, the critical load is calculated by eigenvalue analysis of geometric matrices.

## 2 Modeling of the problem and theoretical formulation

**Fig. 1:**
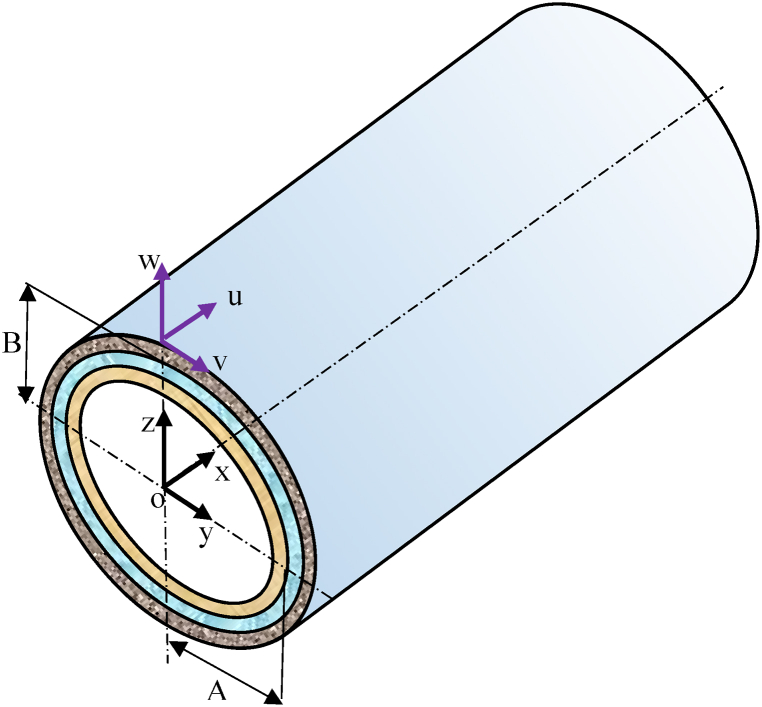
Geometry and coordinate system of an elliptic cylinder.

As schematically demonstrated in Fig. 1, an elastic composite laminate elliptic cylinder is composed of N number of laminas, where the position of each point of the cylindrical shell in the local coordinate systems is indicated with {x,θ,z}. It is also assumed that xε[0,L] $;z\varepsilon \left[-\frac{h}{2},\frac{h}{2}\right]$ θε[0,2π] and the lengths of semi-major and semi-minor axes of the elliptic meridian to be, respectively, A and B. To consider the effects of deformation at each shell cross section, the terms u,v,w are supposed to represent, respectively, longitudinal, tangential, and radial displacements.

It must be noted that for each point of the elliptical cross section, the mean radius r(α), curvature χ(α), and central angel α can be defined as follows [28]:

(1) $$r\left(\alpha \right)=\frac{{\left(A^{2}\;\sin^{2}\;\alpha +B^{2}\;\cos^{2}\;\alpha \right)}^{\frac{3}{2}}}{AB}\;\chi \left(\alpha \right)=\frac{AB}{{\left(A^{2}\;\sin^{2}\;\alpha +B^{2}\;\cos^{2}\;\alpha \right)}^{\frac{3}{2}}}\;\alpha =\mathrm{Arctan}\left(\frac{B}{A}\tan\;\theta \right)$$

### 2.1 Introduction of SFSM

**Fig. 2:**
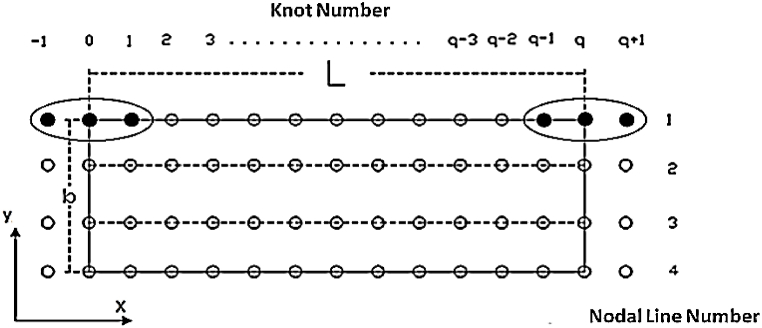
A strip with its knots and nodal lines.

**Fig. 3:**
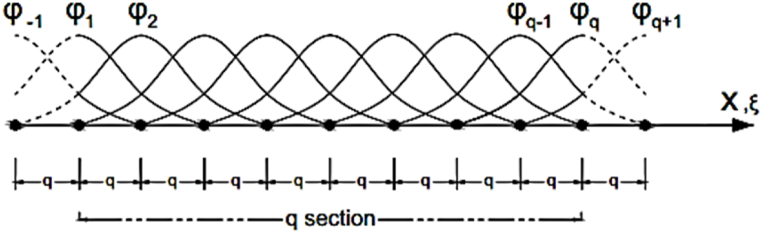
A set of local splines in the longitudinal direction.

The spline function is a mathematical polynomial function named in terms of the degree of function. Spline function can be expressed in the form of B1–B2–B3–B4 spline and B5 spline. Here, cubic B3 spline function is applied to demonstrate the displacements. The B3 spline function (grade 3) is one of the best choices to solve the shell problems, because this function makes it easy to link these problems. Fig. 2 shows a vertical view of a striped element of length L and width b. On the other hand, Fig. 3 reveals how the nodal lines are changed along a strip element. The length of the sheet is divided into n sections (q represents the length of each section), and these divisions are from the points $\varphi_{-1}$ to $\varphi_{q+1}$, with the point $\varphi_{0}$ as the first support and the point $\varphi_{q}$ as the end support of the strip. The two points outside of the longitudinal range of the strip and on the nodal line are used to apply boundary conditions and to modify the spline functions of the boundary areas. In the longitudinal direction, on each nodal line, q+1 nodes numbering from node −1 to node q+1 are used.

**Fig. 4:**
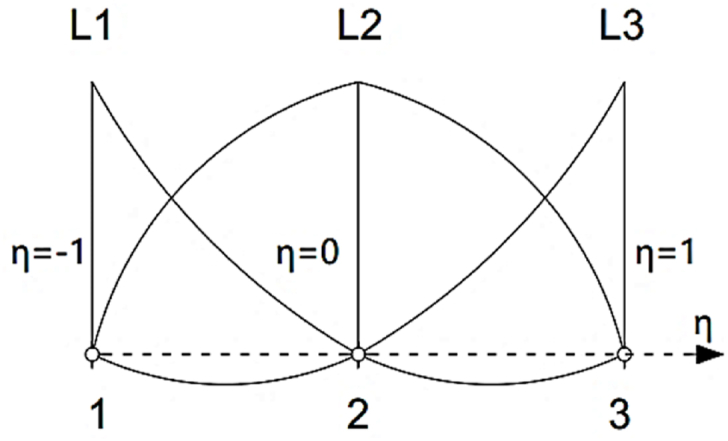
Lagrangian interpolation function in the crosswise direction.

In the crosswise direction of the strip, the Lagrangian functions of degree two with three nodes are used as indicated in Fig. 4, which can be presented as [28]:

(2) $$L_{1}\left(\eta \right)=-\frac{1}{2}\eta \;\left(1-\eta \right)\;L_{2}\left(\eta \right)=\left(1+\eta \right)\left(1-\eta \right)\;L_{3}\left(\eta \right)=\frac{1}{2}\eta \;\left(1+\eta \right)$$

where $L_{j}\left(\eta \right)$ is the term of the shape function relating to the $j^{th}$ nodal line of the strip. Also, η refers to the transverse curvilinear coordinate. However, the general displacement function can be estimated in components of the curvilinear coordinate system using B3-spline and Lagrangian functions as follows [28]:

(3) $$F\left(x,\theta \right)=\sum_{m=1}^{2}\sum_{n=-1}^{q+1}F_{mn}L_{m}^{\left(F\right)}(\theta )\;\varphi_{n}^{\left(F\right)}\left(x\right)$$

in which F expresses the generic generalized displacement function and φ denotes the general term of the B3-spline functions.

According to the FSDT [29], the displacement field $\left(\bar{u},\bar{v},\bar{w}\right)$ for an optional point can be written as [28]:

(4) $$\bar{u}\left(x,\theta ,z\right)=u\left(x,\theta \right)+z\;\psi_{x}\left(x,\theta \right)\;\bar{v}\left(x,\theta ,z\right)=v\left(x,\theta \right)+z\;\psi_{\theta }\left(x,\theta \right)\;\bar{w}\left(x,\theta ,z\right)=w\left(x,\theta \right)$$

where *u*, *v* and *w* refer to the middle surface displacements of shell domain along x, y, and z directions, respectively. Also, $\psi_{x}$ and $\psi_{\theta }$ refer to the normal transverse rotations at the edge of the sheet around the x and θ axes respectively. On the basis of Sanders strain-displacement relations, the linear and nonlinear components of strain-displacement can be defined as [29]:

(5) $$\varepsilon_{x}^{0}=\frac{\partial u}{\partial x}+\frac{1}{2}\left\{C_{3}{\left(\frac{\partial u}{\partial x}\right)}^{2}+C_{3}{\left(\frac{\partial v}{\partial x}\right)}^{2}+{\left(\frac{\partial w}{\partial x}\right)}^{2}\right\}\;\varepsilon_{\theta }^{0}=\frac{\partial v}{\partial \theta }+\frac{w}{R}+\frac{1}{2}\left\{C_{3}{\left(\frac{\partial u}{\partial x}\right)}^{2}+C_{3}{\left(\frac{\partial v}{\partial x}+\frac{w}{R}\right)}^{2}+{\left(\frac{\partial w}{\partial x}-C_{1}\frac{v}{R}\right)}^{2}\right\}\;\gamma_{x\theta }^{0}=\frac{\partial u}{\partial \theta }+\frac{\partial v}{\partial x}+C_{3}\frac{\partial u}{\partial x}∙\frac{\partial u}{\partial \theta }+C_{3}\frac{\partial v}{\partial x}\left(\frac{\partial v}{\partial \theta }+\frac{w}{R}\right)+\frac{\partial w}{\partial x}\left(\frac{\partial w}{\partial \theta }-C_{1}\frac{v}{R}\right)\;k_{x}=\frac{{\partial \psi }_{x}}{\partial x}\;;k_{\theta }=\frac{{\partial \psi }_{\theta }}{\partial \theta }\;k_{x\theta }=\frac{{\partial \psi }_{x}}{\partial \theta }+\frac{{\partial \psi }_{\theta }}{\partial x}+\left\{C_{2}∙\frac{\left(\frac{\partial v}{\partial x}-\frac{\partial u}{\partial \theta }\right)}{2\;R}\right\}\;\gamma_{\theta z}=\frac{\partial w}{\partial \theta }+\psi_{\theta }-C_{1}\frac{v}{R}\;;\;\gamma_{zx}=\frac{\partial w}{\partial x}+\psi_{x}\;\varepsilon_{x}^{NL}=\frac{1}{2}\left\{C_{3}{\left(\frac{\partial u}{\partial x}\right)}^{2}+C_{3}{\left(\frac{\partial v}{\partial x}\right)}^{2}+{\left(\frac{\partial w}{\partial x}\right)}^{2}\right\}\;\varepsilon_{\theta }^{NL}=\frac{1}{2}\left\{C_{3}{\left(\frac{\partial u}{\partial x}\right)}^{2}+C_{3}{\left(\frac{\partial v}{\partial x}+\frac{w}{R}\right)}^{2}+{\left(\frac{\partial w}{\partial \theta }-C_{1}\frac{v}{R}\right)}^{2}\right\}\;\gamma_{x\theta }^{NL}=C_{3}\frac{\partial u}{\partial x}∙\frac{\partial u}{\partial \theta }+C_{3}\frac{\partial v}{\partial x}\left(\frac{\partial v}{\partial \theta }+\frac{w}{R}\right)+\frac{\partial w}{\partial x}\left(\frac{\partial w}{\partial \theta }-C_{1}\frac{v}{R}\right)$$

It should be noted that in Eq. (5) by setting $C_{1}=0,C_{2}=C_{3}=1$, Sander's strain-displacement relationships can be obtained. By taking into account the plane stress assumption, the constitutive stress-strain relationships for a single lamina can be denoted as [29]:

(6) $$\left\{\begin{array}{c} \sigma_{x}\\ \sigma_{\theta }\\ \tau_{x\theta }\\ \tau_{\theta z}\\ \tau_{xz} \end{array}\right\}=\left[\begin{array}{ccccc} {\bar{Q}}_{11} & {\bar{Q}}_{12} & {\bar{Q}}_{16} & 0 & 0\\ {\bar{Q}}_{12} & {\bar{Q}}_{22} & {\bar{Q}}_{26} & 0 & 0\\ {\bar{Q}}_{16} & {\bar{Q}}_{26} & {\bar{Q}}_{66} & 0 & 0\\ 0 & 0 & 0 & {\bar{Q}}_{44} & {\bar{Q}}_{45}\\ 0 & 0 & 0 & {\bar{Q}}_{45} & {\bar{Q}}_{55} \end{array}\right]\left\{\begin{array}{c} \varepsilon_{x}\\ \varepsilon_{\theta }\\ \gamma_{x\theta }\\ \gamma_{\theta z}\\ \gamma_{xz} \end{array}\right\}\;.\;\left\{\sigma \right\}=\left[\bar{Q}\right]\left\{\varepsilon \right\}$$

in which ${\bar{Q}}_{ij}\left(i,j=1,2,6\right)$ refer to the transformed reduced stress -stiffness values and ${\bar{Q}}_{ij}\left(i,j=4,5\right)$ represent the shear-stiffness terms [30]. By integrating stresses through the laminate thickness, the stress resultants can be obtained as [30]:

(7) {NxNθNxθMxMθMxθQθQx}=∫−h2h2{σxσθτxθzσxzσθzτxθτθzτzx}dz=[A11A12A16B11B12B1600A12A22A26B12B22B2600A16A26A66B16B26B6600B11B12B16D11D12D1600B12B22B26D12D22D2600B16B26B66D16D26D6600000000A44A45000000A45A55]{εxεθγxθkxkθkxθγθzγzx}

where $N_{x}$, $N_{\theta }$, $N_{x\theta }$, $Q_{x}$ and $Q_{\theta }$, respectively, are the normal membrane forces, shear membrane forces and shear forces along the thickness per unit length. Also, $M_{x}$, $M_{\theta }$ and $M_{x\theta }$ represent bending moments and twisting moment per unit length. Furthermore, $A_{ij}$ , $B_{ij}$ and $D_{ij}$ refer to the membrane rigidity, bending-membrane rigidity and bending rigidity, which can be denoted as [30]:

(8) $$\left(A_{ij},B_{ij},D_{ij}\right)=\int_{-\frac{h}{2}}^{\frac{h}{2}}\left(1,z,z^{2}\right){\bar{Q}}_{ij}\;dz,i.j=1.2.6\;\left(A_{ij}\right)=k_{i}k_{j}\int_{-\frac{h}{2}}^{\frac{h}{2}}{\bar{Q}}_{ij}\;dz,i.j=4.5$$

in which $k_{i}$ and $k_{j}$ are shear correction coefficients.

### 2.2 Virtual work

The principle of virtual work is employed to obtain equilibrium equations. On the basis of the principle of virtual work, the resultant virtual work exerted by internal and external forces for virtual displacements should be zero as [30]:

(9) $$\delta w={\delta w}^{\mathrm{int}}-{\delta w}^{ext}=0$$

where ${\delta w}^{\mathrm{int}}$ and ${\delta w}^{ext}$ express, respectively, the internal and external virtual works. According to the stress vector σ and virtual strain vector δε, internal virtual work can be presented as

(10) $${\delta w}^{\mathrm{int}}=\int \left[\left(\sigma_{xx}\times {\delta \varepsilon }_{xx}\right)+\left(\sigma_{\theta \theta }\times {\delta \varepsilon }_{\theta \theta }\right)+\left(\tau_{x\theta }\times {\delta \gamma }_{x\theta }\right)+\left(\tau_{xz}\times {\delta \gamma }_{xz}\right)+\left(\tau_{\theta z}\times {\delta \gamma }_{\theta z}\right)\right]\;dV$$

By integration thorough thickness of the strip in Eq. (10), the internal virtual work can be rewritten in terms of generalized stresses strains as

(11) $${\delta w}^{\mathrm{int}}=\int_{0}^{L}\int_{\theta_{1}}^{\theta_{2}}\left[\begin{array}{c} \left(N_{x}\;\delta \varepsilon_{x}^{0}\right)+\left(N_{\theta }\;\delta \varepsilon_{\theta }^{0}\right)+\left(N_{x\theta }\;\delta \gamma_{x\theta }^{0}\right)+\left(M_{x}\;\delta k_{x}\right)+\left(M_{\theta }\;\delta k_{\theta }\right)+\\ \left(M_{x\theta }\;\delta k_{x\theta }\right)+\left(Q_{\theta }\;\delta \gamma_{\theta Z}\right)+\left(Q_{x}\;\delta \gamma_{xZ}\right) \end{array}\right]r\;dx\;d\theta$$

By substituting linear and nonlinear strains in Eq. (11), the linear hardness matrix $\left[K_{E}\right]$ and the geometric hardness matrix $\left[K_{G}\right]$ can be expressed as [30]:

(12) $${\delta w}_{\mathrm{int}}^{l}={\left(\delta \left\{\hat{\Delta }\right\}\right)}^{T}\left[K_{E}\right]\left\{\hat{\Delta }\right\}\;{\delta w}_{\mathrm{int}}^{nl}={\left(\delta \left\{\hat{\Delta }\right\}\right)}^{T}\left[K_{G}\right]\left\{\hat{\Delta }\right\}$$

where $\hat{\Delta }$ is displacements and rotations vector of the shell and $\delta \left\{\hat{\Delta }\right\}$ is virtual displacements and rotations.

The external virtual work exerted by concentrated and distributed out of plane loadings (radial loadings) can be defined as [30]:

(13) $${\delta w}^{ext}=\sum_{i}P_{i}{\delta w}_{i}+\iint_{A}q\left(x,y\right)\delta wdA$$

where $P_{i}$ refer to the concentrated force at point *i* and ${\delta w}_{i}$ indicates the virtual displacement conjugate. Furthermore, *q(x,y)* expresses the radial distributed exerted loading.

### 2.3 Implementing boundary conditions

In the SFSM, as in the finite element method (FEM), it is necessary to correct the functions used in boundary conditions (BCs) to satisfy the displacement and geometric boundary conditions. Correction of the geometric BCs on the edges of the shell is possible by eliminating the related degrees of freedom, and in order to correct the displacement BCs, it is necessary to correct the spline functions at the beginning and end of the shell, that is, the supporting conditions. Assuming that the degree of freedom f (ξ, η) is in the first support (the beginning of the shell), the nodal line numbers 1 and zero is considered. At the first supporting point, the nodal line number 1 is ξ = 0 and η = −1.

(14) $$F\left(0,-1\right)=\frac{1}{6}F_{-11}^{\left(F\right)}+\frac{4}{6}F_{01}^{\left(F\right)}+\frac{1}{6}F_{11}^{\left(F\right)}\;F_{-11}^{\left(F\right)}=C_{-1}\;;\;F_{01}^{\left(F\right)}=C_{0}\;;F_{11}^{\left(F\right)}=C_{1}\;F\left(0,-1\right)=C_{0}\left(\varphi_{0}^{\left(F\right)}-4\varphi_{-1}^{\left(F\right)}\right)+C_{1}\left(\varphi_{1}^{\left(F\right)}-\varphi_{-1}^{\left(F\right)}\right)={C_{0}\left(\varphi_{0}^{\left(F\right)}\right)}_{modified}+{C_{1}\left(\varphi_{1}^{\left(F\right)}\right)}_{modified}$$

According to Eq. (14), the modified spline functions for satisfying F(0,−1) can be expressed as [29]:

(15) $${\left(\varphi_{0}^{\left(F\right)}\right)}_{modified}=\varphi_{0}^{\left(F\right)}-4\varphi_{-1}^{\left(F\right)}\;{\left(\varphi_{1}^{\left(F\right)}\right)}_{modified}=\varphi_{1}^{\left(F\right)}-\varphi_{-1}^{\left(F\right)}\;{\left(\varphi_{-1}^{\left(F\right)}\right)}_{modified}=0\;{\left(\varphi_{q}^{\left(F\right)}\right)}_{modified}=\varphi_{0}^{\left(F\right)}-4\varphi_{q+1}^{\left(F\right)}\;{\left(\varphi_{q-1}^{\left(F\right)}\right)}_{modified}=\varphi_{q-1}^{\left(F\right)}-\varphi_{q+1}^{\left(F\right)}\;{\left(\varphi_{q+1}^{\left(F\right)}\right)}_{modified}=0$$

### 2.4 Analytical integration

**Fig. 5:**
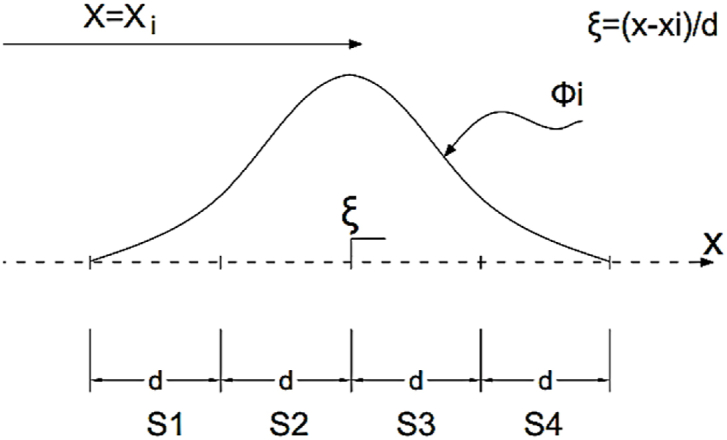
Local spline function in the longitudinal direction.

In some references, for semi-analytic SFSM which uses harmonic functions in the longitudinal direction of the strip, the analytical (Precise) integration has been used [31]. But in the SFSM similar to other numerical methods, so far, numerical integration has been used to solve integral problems [32]. This paper presents a method in which all integrals are calculated analytically and precisely. In this method, because of calculating the integrals before the analysis accurately, the speed of the analysis is increased and the problem solutions can be calculated more accurately. Due to the complexity of calculating the integrals of the spline functions compared to Lagrangian functions, this section discusses how to integrate spline functions accurately. The cubic B-splines in four consecutive sections have non zero values, for example, $\emptyset_{i}$ between knots i-2 to i+2 involve non-zero values (Fig. 5 (.

The polynomials of a B3-spline, named S1, S2, S3 and S4, are given as

(16) $$S_{1}={\frac{1}{6}\left(\xi +2\right)}^{3}\;S_{2}={\frac{1}{6}\left(\xi +2\right)}^{3}-{\frac{4}{6}\left(\xi +1\right)}^{3}\;S_{3}={\frac{1}{6}\left(2-\xi \right)}^{3}-{\frac{4}{6}\left(1-\xi \right)}^{3}\;S_{4}={\frac{1}{6}\left(2-\xi \right)}^{3}$$

on the other hand, all types of integrals involved in forming the tangent stiffness matrix and residual vector can be presented as

(17) $$I_{ij}=\int_{0}^{L}\varphi_{i}\varphi_{j}\;dx\;I_{i^{\prime }j}=\int_{0}^{L}\varphi_{i}^{\prime }\varphi_{j}\;dx\;I_{ij^{\prime }}=\int_{0}^{L}\varphi_{i}\varphi_{j}^{\prime }\;dx\;I_{{i^{\prime }j}^{\prime }}=\int_{0}^{L}\varphi_{i}^{\prime }\varphi_{j}^{\prime }\;dx$$

where $\varphi_{i}^{\prime }=\frac{\partial \varphi_{i}}{\partial x}$. By employing areas $S_{1}$ to $S_{4}$ expressed for each $\varphi_{i}$ or $\varphi_{i}^{\prime }$, it is possible to transform integration over strip length.

**Fig. 6:**
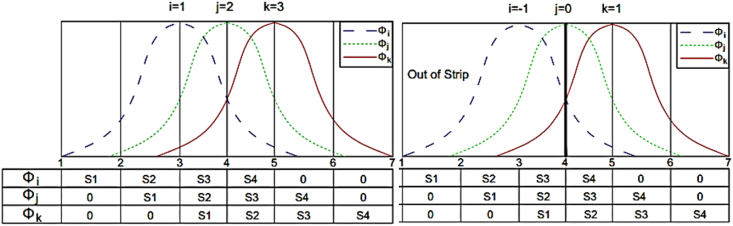
Local spline functions $\emptyset_{i},\emptyset_{j},\emptyset_{k}$ (a) in the domain, (b) near the boundary.

Fig. 6 shows a range of spline functions $\emptyset_{i}$*,* $\emptyset_{j}$ and $\emptyset_{k}$, which have certain values at the three points *i*, j and k in the middle range of the shell (Fig. 6a) as well as for the supporting conditions (Fig. 6b). According to Fig. 6, the common areas between the three spline functions are calculated in three points including two middle and one supporting points as follows:

(18) $$I_{123}=\int_{0}^{L}\varphi_{1}\varphi_{2}\varphi_{3}\;dx=\int_{0}^{d}s_{3}s_{2}s_{1}\;dx+\int_{0}^{d}s_{4}s_{3}s_{2}\;dx\;I_{-101}=\int_{0}^{L}\varphi_{-1}\varphi_{0}\varphi_{1}\;dx=\int_{0}^{d}s_{4}s_{3}s_{2}\;dx$$

It should be noted that in the MATLAB software environment, a subroutine is written to calculate the spline functions integral for estimating the hardness matrices, residuals vector and load vector:

### 2.5 Eigenvalue problem

Finally, the buckling equation can be expressed as eigenvalue problem as [30]:

(19) $$\left|K_{E}-\lambda K_{G}\right|=0$$

in which λ represents the buckling load of the structure, which can be calculated by achieving the non-trivial solution of relation (19) using the subtype repeat method and subroutine which is written in MATLAB software.

## 3 Numerical results and discussion

### 3.1 Verification of results

Before presenting the main results, the correctness and accuracy of the attained formulations must be established. Firstly, the convergence checking of computations is systematically ensured in a standard trial & error manner, i.e., by calculating the number of elements and longitudinal Sections accumulating the series truncation constants, while looking for stability in the predicted buckling load. Hence, in Table 1, the axial critical pressure is calculated under uniform loading of an isotropic elliptical cylindrical shell with radial ratio, λ=0.3 and a length of 200 mm, for N three-node element in crosswise direction and *q* section in longitudinal direction. It is seen that by increasing the number of the elements and longitudinal sections, the buckling load will be more accurate and closer to the buckling factor of the FE. As can be found this table, for 18 or more three-node elements and 20 or more longitudinal sections, the buckling load has a good convergence. Therefore, in order to reduce the analysis time, in the following sections, 18 nodes with 20 longitudinal sections are used.

**Table 1:** Convergence study of the buckling force analysis of the isotropic elliptical shell for L = 200.

Number of element	λ	Number of sections				
		8	10	16	18	20
10	0.3	5.52	5.1	4.83	4.7	4.6
	0.5	7.95	7.87	7.57	7.45	7.18
	1	13.34	13.12	12.94	12.5	12.1
15	0.3	5.37	4.95	4.78	4.5	4.22
	0.5	7.89	7.6	7.4	7.1	7.04
	1	12.96	12.36	12.11	11.88	11.54
20	0.3	5.06	4.63	4.23	4.14	4.11
	0.5	7.31	6.94	6.82	6.6	6.54
	1	12.22	12.09	11.72	11.37	11.29
24	0.3	4.79	4.53	4.2	3.9	3.86
	0.5	7.11	6.76	6.69	6.45	6.39
	1	11.7	11.8	11.65	11.21	11.19

Table 2 presents the buckling load of the shell against the different amounts of the lengths and variable ratio of λ using FSDT. For validation purposes, the results of the spline finite strip analysis are checked with those of previous studies [33] and ABAQUS FE software in Table 2. The area of the elliptical cross section is considered constant and equal to $10^{4}\pi$. The value of the shell thickness is supposed to be 1 mm and the shell length is considered to be variable. However, the shell is supposed to be isotropic with E = 210 GPa, v = 0.3. In Table 2, the buckling load of the SFSM analysis is calculated for the number of cross-sectional elements and longitudinal sections. The ABAQUS FE analysis has been conducted with 7600 S4R5 elements, which are dedicated to thin wall shells and small strains.

**Table 2:** Comparison study of the buckling force analysis of the isotropic elliptical cylindrical shell.

L(mm)	λ	SFSM	FEM	[33]
100	0.3	4.13	4.18	4.28
	0.5	6.59	6.51	6.59
	1	12.03	12.21	12.1
200	0.3	4.14	3.16	4.28
	0.5	6.61	6.66	6.59
	1	11.37	11.49	12.11
300	0.3	4.11	4.12	4.28
	0.5	6.66	6.62	6.59
	1	11.87	11.45	12.10

In another comparison study, the results of the analysis by SFSM for a composite elliptical cylindrical shell are demonstrated in Table 3 and are validated with the results of ABAQUS simulation, when: $E_{11}=176\;GPa,E_{22}=7.04\;GPa,G_{12}=G_{13}=3.52\;GPa\;;\;G_{23}=1.408\;GPa$ and ν=0.25. The effect of layering type and the radial ratio on the critical load of elliptical cylindrical shell has been investigated under a uniform load for the variable length of the shell.

**Table 3:** Critical buckling force of composite cylindrical shell with different layering.

Length	layering sequences	λ=0.3			λ=0.5			λ=1		
		SFSM	FEM	Er (%)	SFSM	FEM	Er (%)	SFSM	FEM	Er (%)
100	${\left(0\right)}_{8}$	41.6	39.9	4.2	60.9	61.1	0.3	94.3	94.7	0.4
	${\left(90\right)}_{8}$	41.1	40.7	0.9	60.3	60.3	0	93.3	93.5	0.2
	${\left(0,90\right)}_{2s}$	74.2	66.9	10.9	114.4	109.8	4.1	165.7	166.7	0.5
	${\left(0,90,90,0\right)}_{s}$	78.8	70	12.5	122.4	111.2	10	165.7	167.4	1.0
200	${\left(0\right)}_{8}$	46.6	40.7	14.4	67.6	69.1	2.1	97.5	94.5	3.1
	${\left(90\right)}_{8}$	39.7	41.6	4.5	60.8	60.8	0	95.2	96.9	1.7
	${\left(0,90\right)}_{2s}$	68.3	66.9	2.0	100.2	96.9	3.4	147	148.9	1.2
	${\left(0,90,90,0\right)}_{s}$	69.2	70.1	1.2	104.1	100.2	3.8	151.7	155.1	2.1

For validation purposes, in different radial ratios and different values of the cylinder length, the buckling force is inferred from the linear analysis of the finite strip eigenvalue and the FE analysis with ABAQUS software and then the results are compared. In ABAQUS analysis, 8000 S4R5 elements are used, while in the finite strip analysis, 18 three-node elements in crosswise direction and 20 longitudinal sections are applied. According to the conducted studies, both analyzes have a good convergence. From the hypothesized layers, the layering mode of ${\left(0\;̗90\;̗90\;̗0\right)}_{s}$ due to the position angle of the layers to the applied load, has more buckling load than single-directional layering modes of ${\left(90\right)}_{8}$ And ${\left(0\right)}_{8}$ and the layering mode of ${\left(0\;̗90\right)}_{2s}$. It is also observed that for any layering mode at different radial ratios, the more elliptical section approaches to the circle, consequently, the more buckling load will be resulted. Also, the buckling load decreases with increasing shell thickness for any material layering

**Fig. 7:**
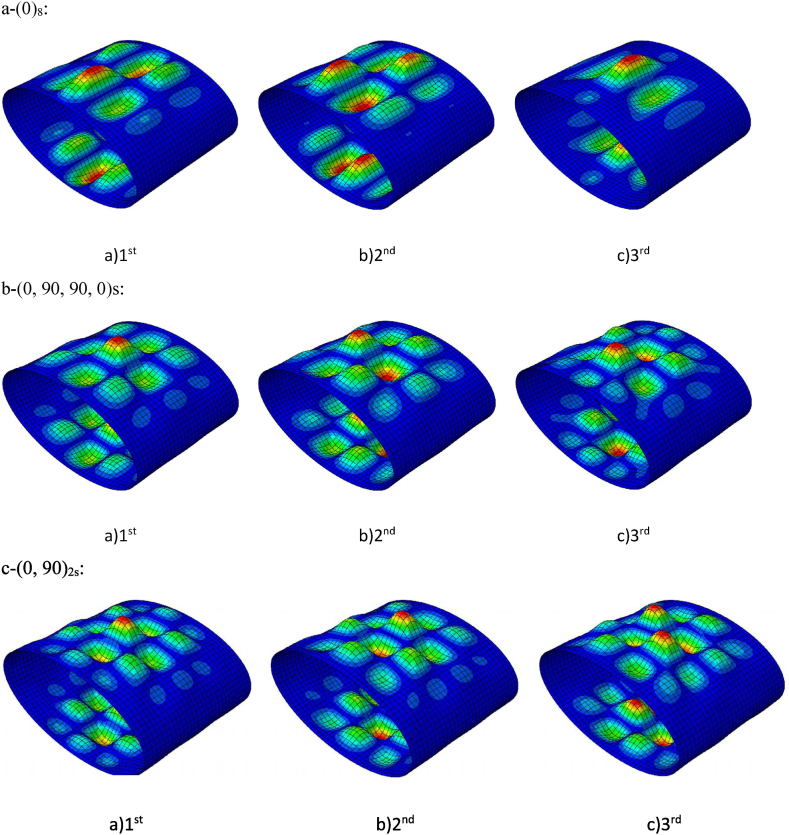
Buckling modes of angle-ply laminated cylinders (L/R = 2.5, the first third modes).

Fig. 7 gives the buckling modes for the radial ratio of λ=0.5 for the layering of ${\left(90/90/0\right)}_{s},{\left(0/90\right)}_{2s}$ and ${\left(0\right)}_{8}$. According to the data obtained from the three-dimensional contours, the most changes and the buckling state of the cylinders happened in the (0,90) state. Hence, the maximum differences in the buckling occurred in the middle of the layers, and it decreased from this value around.

### 3.2 Benchmark results

Numerical computations are performed in current section to better understanding of influences of various terms (different types of layering, high semi ellipse, wide semi ellipse and full ellipse) on the variations of buckling load of laminated composite elliptical shell under non-uniform loading. It is necessary to mention that specifications of non-isotropic materials used in the next sections are: $E_{11}=206.844\;GPa,E_{22}=18.6152\;GPa,G_{12}=G_{13}=4.8162\;GPa,G_{23}=2.551\;GPa,\nu =0.25$ and =100mm,h=1mm.

**Fig. 8:**
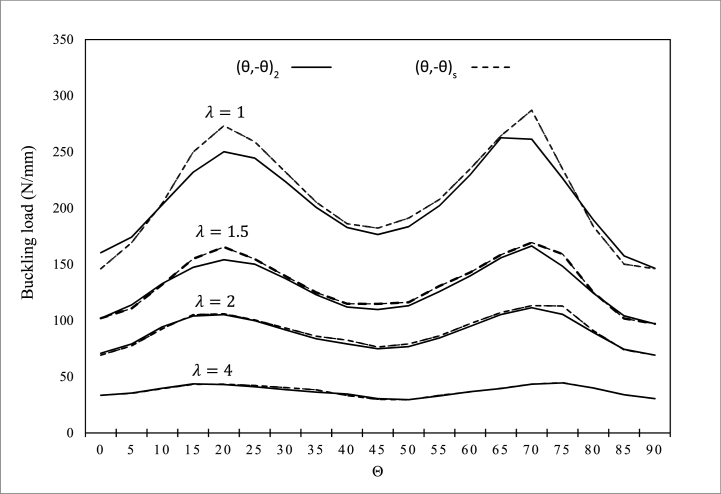
Buckling force of the composite cylindrical shell under the uniform axial force on the elliptical cross section for different radius ratio and the two layering modes of ${\left(\theta \;̗₋\theta \right)}_{s}$ and ${\left(\theta \;̗₋\theta \right)}_{2}$.

**Fig. 9:**
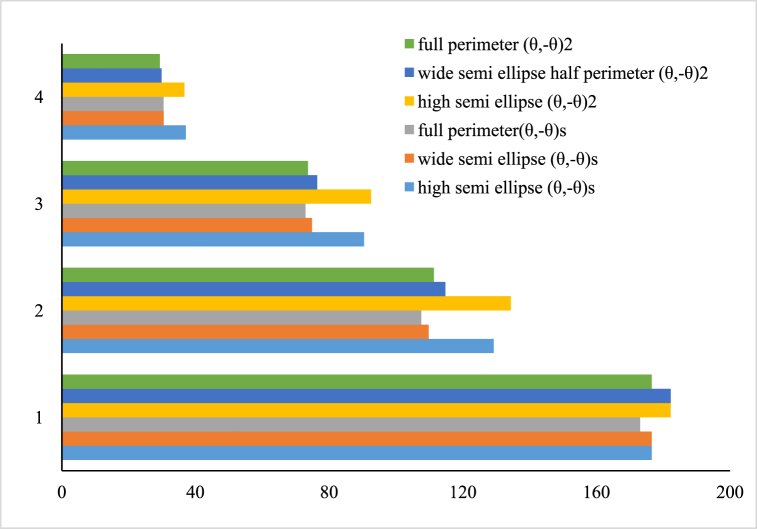
Buckling load of the composite cylindrical shell under non-uniform axial force on the upper half of the ellipse for different radius ratio and the two layering modes of ${\left(\theta \;̗₋\theta \right)}_{s}$ and ${\left(\theta \;̗₋\theta \right)}_{2}$.

In Fig. 8, Fig. 9, the effect of the two types of symmetric and nonsymmetrical layering of ${\left(\theta \;̗₋\theta \right)}_{s}$ and ${\left(\theta \;̗₋\theta \right)}_{2}$ under uniform and non-uniform axial loadings against the various values of radius ratio λ on buckling load is investigated. Fig. 8 will have symmetrical states by increasing the position angle of the layers. For both symmetric and non-symmetrical layering modes of ${\left(\theta \;̗-\theta \right)}_{s}$ and ${\left(\theta \;̗-\theta \right)}_{2}$, under uniform and non-uniform axial loading, the layers have the same behavior with increasing angle. Both layering modes at angle of 45° have the lowest buckling load and in the case of −20- and −70° angles have the highest buckling force. Moreover, according to Fig. 9, the layer mode buckling load of ${\left(\theta \;̗₋\theta \right)}_{2}$ is always higher than the maximum and minimum values of the layer mode buckling force ${\left(\theta \;̗₋\theta \right)}_{s}$. increase. Note that as the λ ratio increases, the curve approaches a horizontal position and the buckling load decreases.

**Fig. 10:**
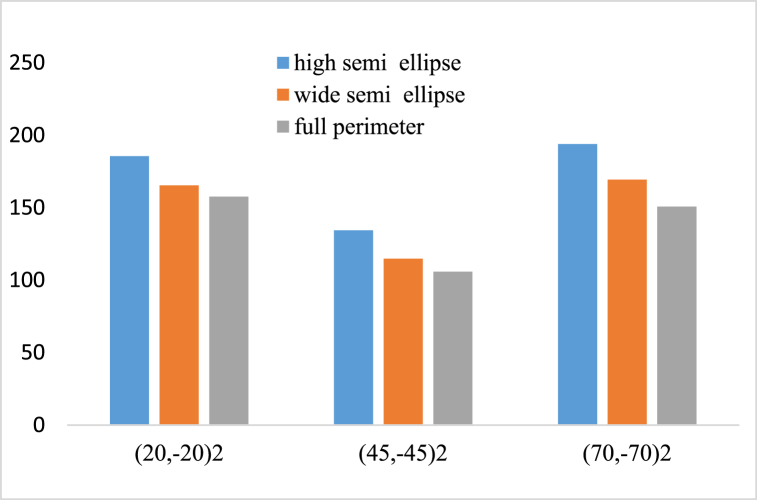
Buckling load of the cylindrical shell for tree axial loading modes on the perimeter of the high semi ellipse, wide semi ellipse and full ellipse.

Fig. 10 reveals that for a constant radius ratio of the cross section λ = 1.5, and for non-symmetrical layering modes of ${\left(\theta \;̗₋\theta \right)}_{2}$, when the load is applied to the parameter of high semi ellipse, the buckling load is higher compared to the states that the load is applied on the perimeter of wide semi ellipse and full ellipse.

## 4 Conclusions

According to the first order shear deformation plate assumption, current paper numerically investigates the buckling problem of cylindrical shells with oval cross-section under simply supported boundary condition subjecting to uniform and non-uniform loads via spline finite strip method. Equilibrium governing equations are generated based upon the virtual work's principle. Shell displacement function is assumed to be as dual development of degree three spline functions and Lagrange polynomial functions. The analysis method used at any time with any type of loading can calculate deformation and buckling. The step-by-step critical load is calculated using the determinant of the stiffness matrix. In the present paper, it is supposed that the applied linear elastic materials consist of isotropic and non-isotropic materials. Numerical studies are performed to indicate the effects of shell geometry, materials, and layered materials on the buckling load. For the validation purpose, the results of current research are compared with those of previous researches and ABAQUS software. The results are as follows:

• In the SFSM considering the very low number of strips, relatively appropriate answers can be achieved in the short time compared to the FEM. This is attributed to the fact that the fewer degrees of freedom than those of the FEM and the separation of integrals in the two directions, which increases the speed of calculating the hardness.
• By keeping the shell volume constant, changing the radius ratio (λ) reduces the buckling force of the composite cylindrical shell. In fact, due to the stretching of the cylindrical section, the structure suffers from a geometric defect which leads to its weakening against the applied load.
• By reducing the axial load area, a lower tension occurs in the shell resulting in hardening of the shell and reducing buckling load. Thus, for a shell with a constant cross section under a non-uniform load on wide semi of ellipse perimeter, the buckling occurs faster compared to the state that non-uniform pressure applies on high semi of the cross-section perimeter.
• According to the data obtained from the three-dimensional contours, the most changes and the buckling state of the cylinders happened in the (0,90) state. Hence, the maximum differences in the buckling occurred in the middle of the layers, and it decreased from this value around.

## Data availability statement

No data availability statement existed in this article.

## Funding statement

No funding has been allocated to this article.

## Author contribution statement

A. Alizadeh: Conceived and designed the analysis. N. Korkeai and P. Pasha: Analyzed and interpreted the data. D. Poorveis and SH. Moradi : Contributed analysis tools or data. N. Korkeai: Wrote the paper.

## Declaration of competing interest

The authors declare that they have no known competing financial interests or personal relationships that could have appeared to influence the work reported in this paper.
